# CRISPR/Cas9-mediated knock-in cells of the late-onset Alzheimer’s disease-risk variant, SHARPIN G186R, reveal reduced NF-κB pathway and accelerated Aβ secretion

**DOI:** 10.1038/s10038-024-01224-x

**Published:** 2024-02-13

**Authors:** Yuya Asanomi, Tetsuaki Kimura, Nobuyoshi Shimoda, Daichi Shigemizu, Shumpei Niida, Kouichi Ozaki

**Affiliations:** 1 Medical Genome Center, Research Institute, National Center for Geriatrics and Gerontology, Obu, Aichi Japan; 2 RIKEN Center for Integrative Medical Sciences, Yokohama, Japan; 3 Center for Core Facility Administration, Research Institute, National Center for Geriatrics and Gerontology, Obu, Aichi Japan

**Keywords:** Genetic engineering, Rare variants, Alzheimer's disease, Mutagenesis, Alzheimer's disease

## Abstract

Despite recent great successes in identifying many novel genetic variants associated with late-onset Alzheimer’s disease (LOAD), the direct biological relevance of these variants to the disease is largely unresolved. In our previous report, we identified a rare functional variant of *SHARPIN*, rs572750141 (G186R), that is significantly associated with LOAD. Other missense variants of *SHARPIN* have been found to be associated with LOAD susceptibility in recent large-scale meta-analyses of genome-wide association studies. Although functional analyses of the G186R-type SHARPIN protein in previous studies have revealed aberrant cellular localization of this protein and attenuated activation of the NF-κB pathway, all these analyses used exogenous gene transfer. We used the CRISPR/Cas9 system to perform a knock-in of the LOAD-risk variant into HEK293 cells and generated cell lines homozygous for the SHARPIN G186R mutation. Although the efficiency of the knock-in was modest (<1%), the desired knock-in cells were successfully obtained through high-throughput screening by using a PCR-Invader assay. In the G186R knock-in cells, TNF-α-induced activation of the NF-κB pathway was strongly suppressed, but aberrant cellular localization of the mutant protein was not apparent. Furthermore, the amounts of amyloid-β (Aβ) 40 and 42 secreted into the culture medium were significantly increased in the G186R knock-in cells, although no significant change in the ratio of Aβ40 to Aβ42 was observed. These findings from the knock-in cells indicate the effect of the LOAD-risk variant on SHARPIN functions more directly than in previous studies. Further investigation of SHARPIN-related pathways may elucidate the mechanism underlying the onset of LOAD.

## Introduction

Late-onset Alzheimer’s disease (LOAD) is the most common form of dementia. LOAD is a multifactorial disease caused by complicated interactions among multiple environmental and genetic factors. The heritability of LOAD was estimated to be high (*h*^2^ = 58–79%) in a large twin study [1]. *APOE* ε4 is the most important genetic risk factor, and the latest meta-analysis of genome-wide association studies (GWAS) based on large Caucasian cohorts reported 75 risk loci associated with Alzheimer’s disease and related dementias [2]. A large proportion of the heritability, however, remains unexplained. In our previous study, we conducted whole-exome sequencing analyses of 202 Japanese LOAD patients who were negative for the *APOE* ε4 risk allele [3]. Through the analysis, we identified a rare functional variant of *SHARPIN*, rs572750141 (p.Gly186Arg), that was significantly associated with an increased risk of LOAD pathogenesis in a large Japanese cohort consisting of 4563 LOAD patients and 16,459 controls (odds ratio = 6.1).

SHARPIN (SHANK-associated RH domain interactor) is a multifunctional protein associated with numerous physiological processes and a variety of diseases. SHARPIN forms linear ubiquitination assembly complex (LUBAC), which regulates the activation of the nuclear factor kappa B (NF-κB) pathway, a central player in immune and inflammatory responses [4–10]. Additionally, SHARPIN has other physiological roles in various processes, such as tumor necrosis factor-α (TNF-α)-induced cell death [11], regulation of caspase 1 activity in sepsis [12], and the progression of many types of cancers [13–20]. Recent large GWAS meta-analyses of LOAD have discovered numerous novel risk loci, among which are missense variants of *SHARPIN* [2, 21]. Therefore, the functional role of SHARPIN in the development of LOAD has been attracting attention. In our previous studies, we performed functional analyses of LOAD-risk variant-type SHARPIN protein, and we found aberrant cellular localization of the variant protein and attenuated activation of the NF-κB pathway [3, 22]. These studies, however, were not performed under physiological conditions. Therefore, the results of the experiments were limited because the presence of endogenous wild-type SHARPIN may have led to aberrant synergic effects, and the higher level of overexpressed SHARPIN protein than of the endogenous protein may have influenced other physiological cellular functions or activities. To elucidate the role of LOAD-risk variants in disease pathogenesis, there is a need for more direct ways of examining the function of mutant SHARPIN protein.

In this study, we investigated the function of a SHARPIN mutation in a more physiological way by using knock-in cells generated through genome editing techniques. The CRISPR/Cas9 system with donor oligo DNA enables knock-in of the target single nucleotide variant (SNV) into cultured cell lines. We performed knock-in of an SNV, rs572750141 (SHARPIN G186R), which we previously identified as a LOAD risk variant with strong effect size [3], into HEK293 cells, and we obtained cells homozygous for the mutation by single-cell cloning. We then used the knock-in cell line to examine the intracellular localization of endogenous SHARPIN protein and the TNF-α-induced activation of the NF-κB pathway. We also compared extracellular amyloid-β (Aβ) concentrations in wild-type and G186R knock-in cells. Clarification of the effect of the LOAD-risk variant on SHARPIN function may help to elucidate the mechanisms of SHARPIN in LOAD pathogenesis.

## Materials and methods

### Oligonucleotides

To perform genome editing with the CRISPR/Cas9 system, we designed Alt-R CRISPR-Cas9 crRNA (crRNA) and Alt-R HDR Donor Oligo (donor ssDNA) by using the Alt-R HDR Design Tool (Integrated DNA Technologies, Coralville, IA, USA). The sequences of crRNA and donor ssDNA are provided in Supplementary Table S1. The designed crRNA, donor ssDNA, and a universal tracrRNA (trans-activating crRNA) were purchased from Integrated DNA Technologies. Primers for PCR, Sanger sequencing, and Invader assays were commercially synthesized (Fasmac, Kanagawa, Japan).

### Transfection with gRNA, Cas9 protein, and donor ssDNA

HEK293 cells were plated at a density of 3 × 10^5^ cells/well in 6-well plates and cultured in DMEM for 24 h. Guide RNA (gRNA) was prepared by mixing equimolar amounts of crRNA and tracrRNA and heating to 95 °C for 5 min, followed by slow cooling to room temperature. Transfection with gRNA (final concentration 0.43 µg/mL), Alt-R S.p. Cas9 Nuclease V3 (Integrated DNA Technologies) (final concentration 2.24 µg/mL), and donor ssDNA (final concentration 2.93 nM) was performed simultaneously by using Lipofectamine CRISPRMAX (Thermo Fisher Scientific, Waltham, MA, USA) in accordance with the manufacturer’s protocol.

### Single-cell cloning

Twenty-four hours after the transfection, the cells were plated at a density of 2 cells/well on 96-well plates and cultured in DMEM for 13 to 17 days until sufficient cell growth was achieved. The day before we checked for clones by genotyping, the cells were suspended in the culture medium and half of them were aliquoted into new 96-well plates. The aliquoted plates were used for subsequent genotyping and sequencing analyses.

### PCR from cultured cells

Cells cultured on 96-well plates were washed with PBS before genomic DNA extraction. The cells were then suspended in 20 µL of RIPA buffer (Merck, Darmstadt, Germany) and diluted 10-fold with pure water. PCR was performed by using KOD -Multi & Epi- (Toyobo, Osaka, Japan).

### Genotyping

PCR products from the cultured clones were diluted 10-fold with pure water and aliquoted into 384-well plates. Genotyping of each clone was conducted by using an Invader assay (Third Wave Technologies, Madison, WI, USA) [23] and the QuantStudio 7 Flex Real-Time PCR System (Thermo Fisher Scientific). To account for the introduction of silent mutations, primary probes corresponding to alternative alleles were used by mixing equal amounts of those probes with and without silent mutations. To validate the candidate clones identified by the Invader assay, purified PCR products were subjected to Sanger sequencing by using a BigDye Terminator v3.1 Cycle Sequencing Kit and an ABI 3500xL Genetic Analyzer (Thermo Fisher Scientific).

### Immunocytochemistry

Cells were plated at a density of 2.0 × 10^4^ cells/well on BioCoat Poly-D-Lysine 4-well Culture Slides (Corning, Corning, NY, USA) and cultured in DMEM for 24 h. The cells were then fixed with 4% paraformaldehyde (Nacalai Tesque, Kyoto, Japan) and incubated with Rabbit Anti-SHARPIN Polyclonal antibody (dilution 1:200; cat. no. 14626-1-AP; Proteintech, Rosemont, IL, USA) in 2% FBS/PBS for 1 h. This was followed by incubation with Alexa Fluor 488–labeled Goat Anti-Rabbit IgG H&L (dilution 1:500; cat. no. ab150077; Abcam, Cambridge, UK) for 1 h. The slides were mounted by using SlowFade Diamond Antifade Mountant with DAPI (Thermo Fisher Scientific). Fluorescence images were obtained by using a BIOREVO BZ-X800 fluorescence microscope (Keyence, Osaka, Japan).

### Luciferase assay

Cells were transfected with the luciferase reporter plasmid pGL4.32[luc2P/NF-κB-RE/Hygro] (Promega, Madison, WI, USA), and stably expressing cells were selected with hygromycin. Before the luciferase assay, cells were plated on 96-well plates at a density of 1.5 × 10^4^ cells/well and cultured in DMEM for 24 h. The cells were then treated with 20 ng/mL of TNF-α (Wako, Osaka, Japan) for 5 h. Luciferase activity was measured by using the Nano-Glo Dual-Luciferase Reporter Assay System (Promega). Each experiment was independently conducted three times, with five replicates for each sample.

### Aβ ELISA

We used a previously constructed APPswe plasmid [24], which contains human APP695 with the Swedish (KM595/596NL) mutation cloned into pcDNA3.1/Hygro(+) vector (Thermo Fisher Scientific). Cells were transfected with the APPswe plasmid, and stably expressing cells were selected by using hygromycin. Before the ELISA, cells were plated on 6-well culture plates at a density of 4.0 × 10^5^ cells/well and cultured in DMEM for 24 h. We used a human β-amyloid ELISA kit (Wako) to measure the amounts of Aβ40 and Aβ42 secreted into the culture medium. Each experiment was independently conducted three times, with 12 replicates for each sample. The ratio of Aβ42 to Aβ40 was calculated on the basis of the average of the replicates.

## Results

### Construction of SHARPIN G186R knock-in cells by using the CRISPR/Cas9 system

**Fig. 1:**
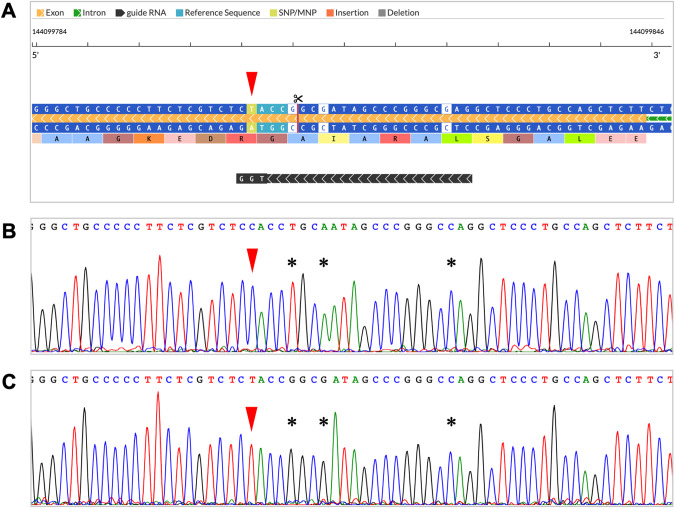
Construction of SHARPIN G186R knock-in cells. **A** Schematic representation of the Alt-R CRISPR-Cas9 crRNA designed by using the Alt-R HDR Design Tool (black bar). Red arrowhead indicates the target SNV in Alt-R HDR Donor Oligo. Details of the designed crRNA and donor ssDNA are given in Supplementary Table S1. Sanger sequencing of the target-flanking region in HEK293 cells (**B**) and knock-in cells (**C**). Red arrowhead indicates the position of the target SNV and asterisks indicate the positions of silent mutations

Knock-in of the target SNV (rs572750141, NP_112236.3:p.Gly186Arg) into HEK293 cells was performed by using the CRISPR/Cas9 system. To prevent recutting of the edited site, donor ssDNA with three silent mutations was designed (Fig. 1A and Supplementary Table S1). Transfected cells were isolated by single-cell cloning using limiting dilution and screened by PCR-Invader assay genotyping. The flanking sequences were confirmed by Sanger sequencing, and the knock-in of the target SNV was verified. As a result, most of the candidate clones had unwanted InDels in one or both alleles. Although the introduction efficiency of the target mutation was low (<1%), high-throughput screening by using the Invader assay was successfully performed, and clones homozygous for knock-in of the target SNV were obtained (Fig. 1B, C). As shown in Fig. 1C, the most distant silent mutation (+19 bp from the target SNV) was not introduced in the clone we used in this study. This was likely due to the recombination repair between the second and third silent mutations.

### Effect of G186R mutation on cellular localization of endogenous SHARPIN protein

**Fig. 2:**
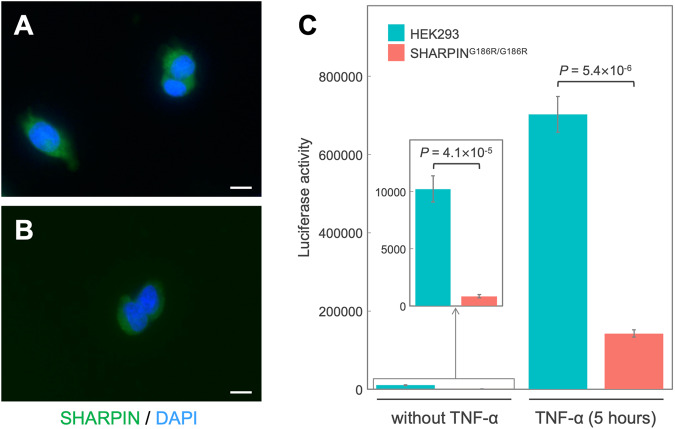
Effect of G186R knock-in on endogenous SHARPIN function. Localization of endogenous wild-type SHARPIN in HEK293 cells (**A**) and of G186R-type SHARPIN in knock-in cells (**B**). SHARPIN was visualized via immunocytochemistry. Scale bars, 10 µm. **C** NF-κB activity with and without TNF-α-induced activation was determined via luciferase assay, which was performed three times with five replicates in each assay. Shown is a representative result from the three assays. The other results are shown in Supplementary Fig. S1

The generated knock-in cells expressing mutant proteins were used to investigate the effects of mutations on the endogenous protein. In a previous study, we found that overexpression of G186R-type SHARPIN resulted in aberrant cellular localization of the SHARPIN protein [3]. Therefore, we first examined the cellular localization of endogenous SHARPIN protein. Endogenous SHARPIN G186R was uniformly distributed throughout the cytosol, similar to wild-type SHARPIN protein (Fig. 2A, B). Although overexpressed mutated SHARPIN was present as uneven clumps of granules [3, 22], it did not colocalize with various organelle markers (data not shown), suggesting that the overexpressed protein was likely aggregating. The endogenous SHARPIN G186R may have not aggregated because its protein level was lower than that in the overexpression experiments.

### SHARPIN G186R reduces TNF-α-induced activation of NF-κB

We investigated the effect of G186R knock-in on TNF-α-induced activation of the NF-κB pathway, a key physiological function of SHARPIN. To measure NF-κB activity in the knock-in cells, we performed luciferase assays by using a reporter plasmid containing an NF-κB response element. In HEK293 cells, NF-κB was activated more than 50-fold in response to TNF-α stimulation (Fig. 2C). Even in the steady state without TNF-α stimulation, NF-κB activity was significantly lower in G186R knock-in cells than in wild-type HEK293 cells (Fig. 2C and Supplementary Fig. S1A, C; *P*-values in three independent assays from a single clone: Welch’s *t*-test *P* = 4.1 ×10^–5^, 4.2 ×10^–7^, 2.2 ×10^–5^). Under TNF-α stimulation, the activation of NF-κB was also dramatically suppressed in knock-in cells (Fig. 2C and Supplementary Fig. S1B, D; *P*-values in three independent assays from a single clone: Welch’s *t*-test *P* = 5.4 ×10^–6^, 1.7 ×10^–5^, 2.0 ×10^–7^). These results corroborate the findings of our previous study performing the overexpression of G186R-type SHARPIN [3, 22].

### Secretion of both Aβ40 and Aβ42 is elevated by SHARPIN G186R

**Fig. 3:**
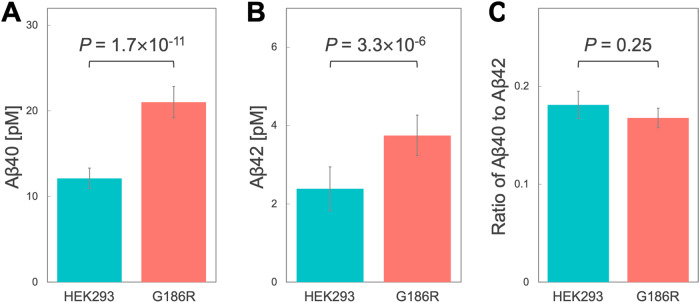
Amounts of Aβ secreted from G186R knock-in cells. Amounts of Aβ40 (**A**) and Aβ42 (**B**) secreted from HEK293 cells and knock-in G186R cells. The experiment was performed three times, with 12 replicates in each assay. Shown are representative results from the three assays. The other results are shown in Supplementary Fig. S2. **C** The ratio of Aβ42 to Aβ40 was calculated from the ELISA results

Brain Aβ accumulation plays a significant role in the pathogenesis of Alzheimer’s disease [25]. NF-κB signaling pathway facilitates the processing of APP and the production of Aβ [26, 27], and Aβ induces NF-κB [28–30]. Moreover, recent findings by Krishnan et al. suggest that SHARPIN is involved in the clearance and degradation of Aβ [31, 32]. To investigate the relationship between SHARPIN mutation and Aβ metabolism, we utilized an Alzheimer’s disease model HEK293 cell line (HEK-APPswe) widely employed in Aβ secretion studies [24, 33–35]. Then, we determined the amounts of Aβ40 and Aβ42 secreted into the extracellular fluid using ELISA. The amounts of Aβ40 and Aβ42 secreted into the culture medium were significantly greater in knock-in cells than in wild-type cells (Fig. 3A, B and Supplementary Fig. S2; *P*-values in three independent assays from a single clone for Aβ40: Welch’s *t*-test *P* = 1.7 ×10^–11^, 4.2 ×10^–7^, 7.5 ×10^–9^; and for Aβ42: Welch’s *t*-test *P* = 3.3 ×10^–6^, 8.1 ×10^–6^, 6.0 ×10^–3^). In contrast, there was no significant change in the ratio of Aβ40 to Aβ42 (Fig. 3C, Welch’s *t*-test *P* = 0.25).

## Discussion

We investigated the effects of the LOAD-risk variant, rs572750141 (G186R), on SHARPIN function more physiologically than in our previous studies. By using the CRISPR/Cas9 system and high-throughput screening with a PCR-Invader assay, we successfully constructed targeted knock-in cell lines homozygous for the SHARPIN G186R mutation. We found that TNF-α-induced NF-κB activation was dramatically suppressed in G186R knock-in cells, whereas the localization of endogenous SHARPIN G186R protein was similar to that of the wild-type protein (Fig. 2 and Supplementary Fig. S1). To further evaluate the allelic effect of the G186R mutation, we confirmed that comparable results were obtained using another homozygous clone (Supplementary Fig. S4). Interestingly, knock-in of G186R caused an increase in the amounts of Aβ secreted into the culture medium (Fig. 3 and Supplementary Fig. S2). Increased extracellular secretion of Aβ could be a risk for LOAD. These findings should contribute to a better understanding of the molecular pathology of LOAD.

The activity of NF-κB was suppressed in G186R knock-in cells compared to wild-type cells, although TNF-α-induced activation of NF-κB occurred in both cells (Fig. 2C and Supplementary Fig. S1). TNF-α-induced activation of NF-κB was impaired, but not completely abolished, in SHARPIN-deficient cells [4–6]. These results imply that the G186R mutation also impairs the SHARPIN function, similar to SHARPIN deficiency. Meanwhile, in the absence of TNF-α stimulation, there was no significant difference in NF-κB activity between cells with wild-type SHARPIN and those with G186R-type SHARPIN in our previous experiments under overexpression conditions (Supplementary Fig. S3). This may have been due to the presence of endogenous wild-type SHARPIN in the overexpression system. Even without stimulation, a low-level signal in the NF-κB pathway was present; we considered that it was maintained by the endogenous wild-type SHARPIN. In contrast, homozygous knock-in resulted in the absence of functional wild-type SHARPIN, thus significantly reducing steady-state NF-κB activity (Fig. 2C and Supplementary Fig. S1A, C).

In the TNF-α-induced NF-κB pathway, SHARPIN forms heterotrimeric LUBAC and regulates downstream signals through linear ubiquitination [4–10]. The latest LOAD-GWAS meta-analysis identified *RBCK1*, which encodes HOIL1 (one of the components of LUBAC), and *OTULIN*, which encodes the LUBAC-associated deubiquitinase OTULIN, as risk genes prioritized with high confidence [2]. Therefore, the importance of the NF-κB pathway through LUBAC formation in LOAD pathogenesis has been suggested. We attempted to assess the impact of the SHARPIN mutation on LUBAC stability and its association with attenuated NF-κB activity. The binding capacity of SHARPIN to HOIL1 and HOIP (another LUBAC component) was evaluated through a co-immunoprecipitation assay. However, G186R-type SHARPIN appeared to be uniformly distributed in the cytoplasm by immunocytochemistry, whereas it exhibited instability in the soluble fraction of the cell lysate, likely forming aggregates, making extremely difficult of quantitative analysis. Recently, Sato et al. [36] clarified the impact of the G186R mutation from the perspective of structural biology in their review, and they predicted reduced interaction between SHARPIN and HOIL1. They also speculated that glycine at this position in the crystal structure of mouse LUBAC is located in the turn region that connects two helices and that the structure is destabilized by dihedral angle restrictions with residues other than glycine. Thus, the G186R mutation may affect signaling in the NF-κB pathway by preventing the stable formation of LUBAC. In the field of LOAD study, it has been suggested that neuroinflammation mediated by microglia and astrocytes in the central nervous system plays an important role in the onset of the disease [37–39]. The attenuated NF-κB activity caused by the SHARPIN G186R mutation may increase the risk of LOAD onset by altering inflammatory and immune responses in the central nervous system.

Although we observed increased secretion of Aβ into the culture medium in knock-in cells (Fig. 3 and Supplementary Fig. S2), it is unclear whether this increase was due to increased Aβ production or to suppression of Aβ degradation. More precise investigations are required to ascertain the involvement of NF-κB in this pathway. Krishnan et al. [31] demonstrated that the knockdown of SHARPIN in THP-1-derived macrophages significantly reduced Aβ phagocytosis. Further insights into the role of SHARPIN in LOAD might be obtained from successful knock-in to macrophages or microglial cells, although we chose HEK293 cells for this study because of the technical challenges of SNV knock-in to cultured cells. The easy-to-transfect immortalized HEK293 cell line permits high-throughput assays and is useful for the study of neurodegenerative diseases [40]. In recent years, there has been an increased focus on the use of anti-amyloid drugs to treat Alzheimer’s disease by removing accumulated Aβ in the brain. However, the potential for anti-Aβ therapies to accelerate brain atrophy has also been suggested [41]. Treatment of deficiencies in the Aβ clearance pathway, including those caused by mutant SHARPIN, may become a novel target for drug discovery. Elucidating the mechanism by which SHARPIN mutation is involved in Aβ secretion or clearance, or both, may provide insights into pharmaceutical approaches to the development of new therapies for LOAD.

The present study has some limitations. The G186R mutation was identified through an analysis of Japanese LOAD patients and is rarely found in individuals outside East Asia. This variant is rare, with a minor allele frequency of less than 0.1% in East Asia, and it is associated with a risk of LOAD with a dominant mode of inheritance. This suggests that the wild-type SHARPIN from another allele may still be functional, resulting in a weaker effect than we observed here. Additionally, we suggested previously that aberrant cellular localization of the overexpressed G186R-type SHARPIN caused the reduction in NF-κB activity. Our failure to find aberrant cellular localization does not seem consistent with our earlier hypothesis. Nonetheless, the findings in the overexpression experiment suggest that G186R-type SHARPIN may be prone to aggregation, and we cannot rule out that it may aggregate as a result of long-term turnover of the protein in differentiated nerve cells of the elderly or by transient protein-level elevation upon stimulation by, for example, extracellular Aβ and inflammation. Thus, the results of this study using HEK293 cells have some limitations. Future investigations focusing on the effects of knock-in of the SHARPIN mutation into differentiated cells of the nervous system, such as microglia, might elucidate further pathological conditions and provide more appropriate insights for the pathogenesis.

In conclusion, we used CRISPR/Cas9-mediated knock-in cells to investigate the effects of the LOAD-risk variant, SHARPIN G186R, on endogenous SHARPIN function. Elucidation of the association between the reduced NF-κB pathway activity caused by the SHARPIN mutation and LOAD pathogenesis may provide clues to a better understanding of LOAD pathogenesis and for innovative pharmaceutical investigations. Further clarification of the role of SHARPIN in Aβ-related pathways may shed light on the mechanism of LOAD onset. With the growing number of LOAD patients, the burden of the disease is increasing, not only for these patients but also for their families and caregivers. Therefore, the development of effective LOAD prevention and treatment approaches is an urgent medical issue. Clarifying the role of SHARPIN in LOAD pathogenesis may lead to the discovery of novel molecular targets for innovative biological and pharmacological approaches in future precision medicine.

### Supplementary information

Table S1

Figure S1

Figure S2

Figure S3

Figure S4

## Data Availability

The datasets analyzed during this study are available from the corresponding author on reasonable request.
