# Supplementary material for: CRISPR/Cas9-mediated knock-in cells of the late-onset Alzheimer’s disease-risk variant, SHARPIN G186R, reveal reduced NF-κB pathway and accelerated Aβ secretion
Source: J Hum Genet. 2024 Feb 13;69(5):171–6. doi: 10.1038/s10038-024-01224-x (PMC11043039; doi:10.1038/s10038-024-01224-x)
Supplement: Supplementary file 1 — Table S1 [file 10038_2024_1224_MOESM1_ESM.pdf]

Table S1. Details for designed crRNA and donor ssDNA.

Product	Sequence	Bases	Tm
Alt-R HDR Donor Oligo	/Alt-R-HDR1/G*C*C AGG ACG GCT GCC ACT TGG GCT GCC CCC TTC TCG TCT CTA CCG GCG ATA GCC CGG GCG AGG CTC CCT GCC AGC TCT TCT GC*A* G/Alt-R-HDR2/	85	77.0
Alt-R∃ CRISPR-Cas9 crRNA	/AltR1/rCrUrG rGrCrC rCrGrG rGrCrU rArUrU rGrCrA rGrGrG rUrUrU rUrArG rArGrC rUrArU rGrCrU /AltR2/	36	64.7
