# Supplementary figures and images for: CRISPR/Cas9-mediated knock-in cells of the late-onset Alzheimer’s disease-risk variant, SHARPIN G186R, reveal reduced NF-κB pathway and accelerated Aβ secretion
Source: J Hum Genet. 2024 Feb 13;69(5):171–6. doi: 10.1038/s10038-024-01224-x (PMC11043039; doi:10.1038/s10038-024-01224-x)

**Figure S1. The other two of three independent assays of the luciferase assay.**

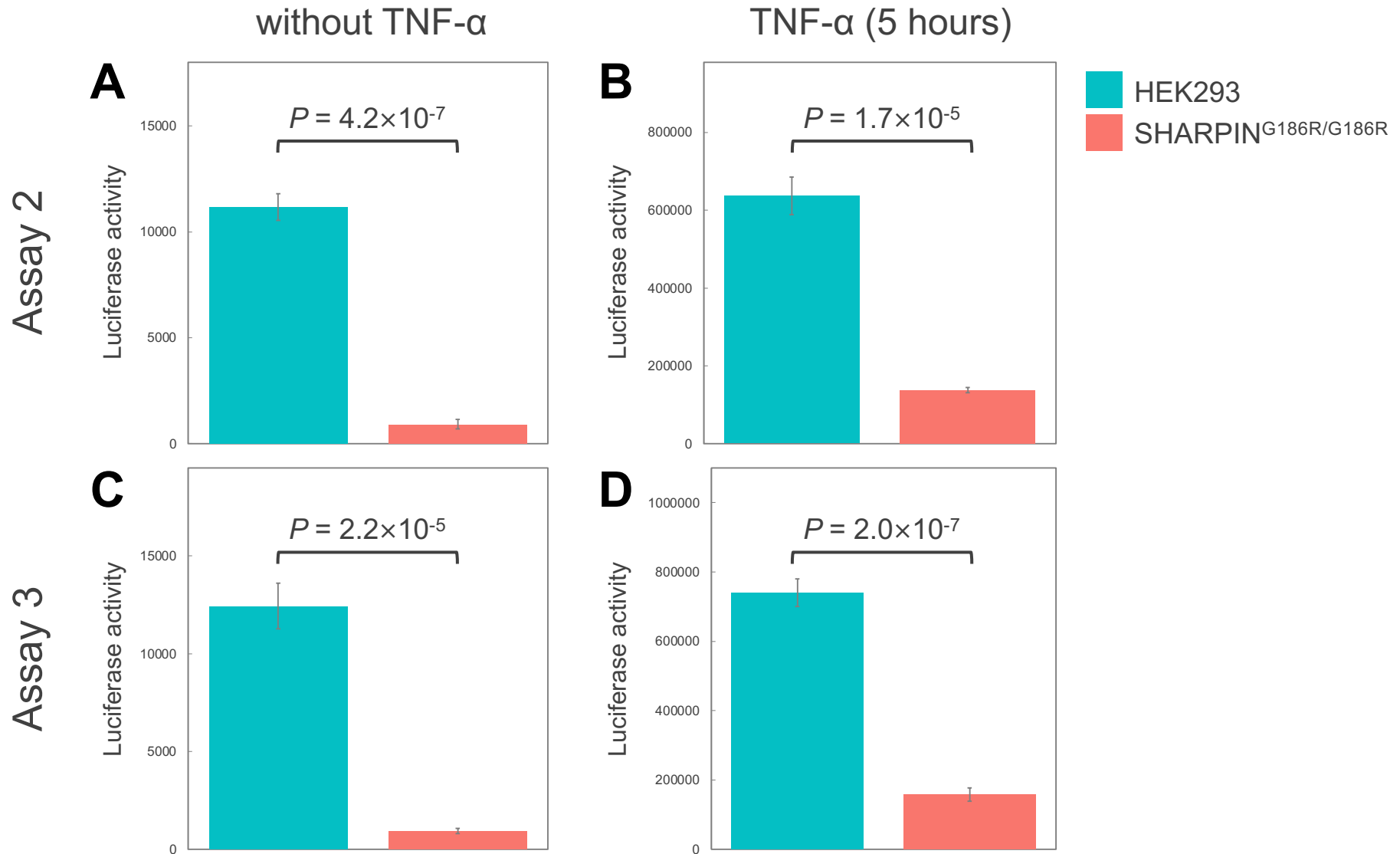

Supplement: Supplementary file 2 — Figure S1 [file 10038_2024_1224_MOESM2_ESM.pdf]

**Figure S2. The other two of three independent assays of A $\beta$  ELISA assay.**

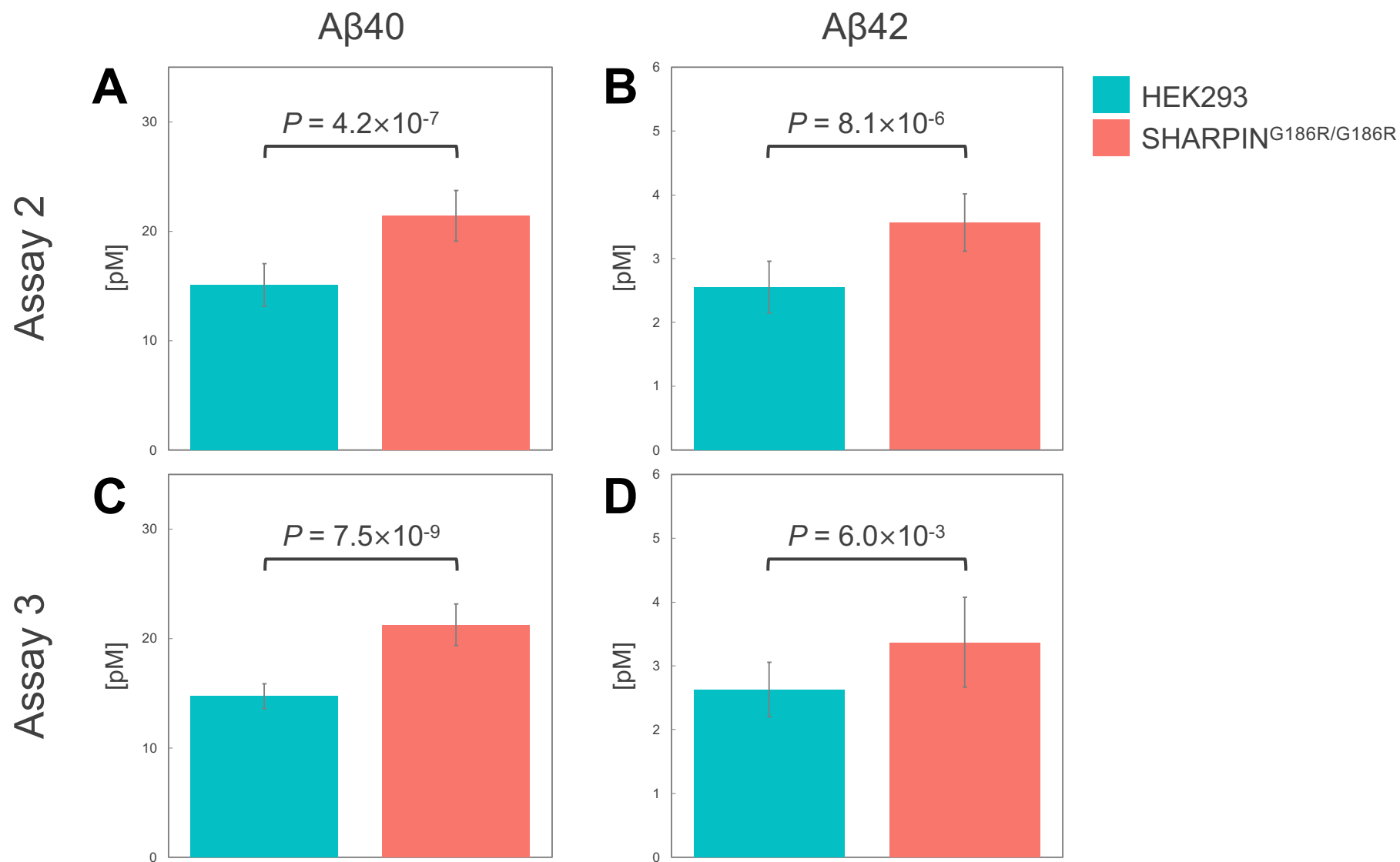

Supplement: Supplementary file 3 — Figure S2 [file 10038_2024_1224_MOESM3_ESM.pdf]

**Figure S3. The NF- $\kappa$ B activity under the overexpression of wild-type and G186R-type SHARPIN.**

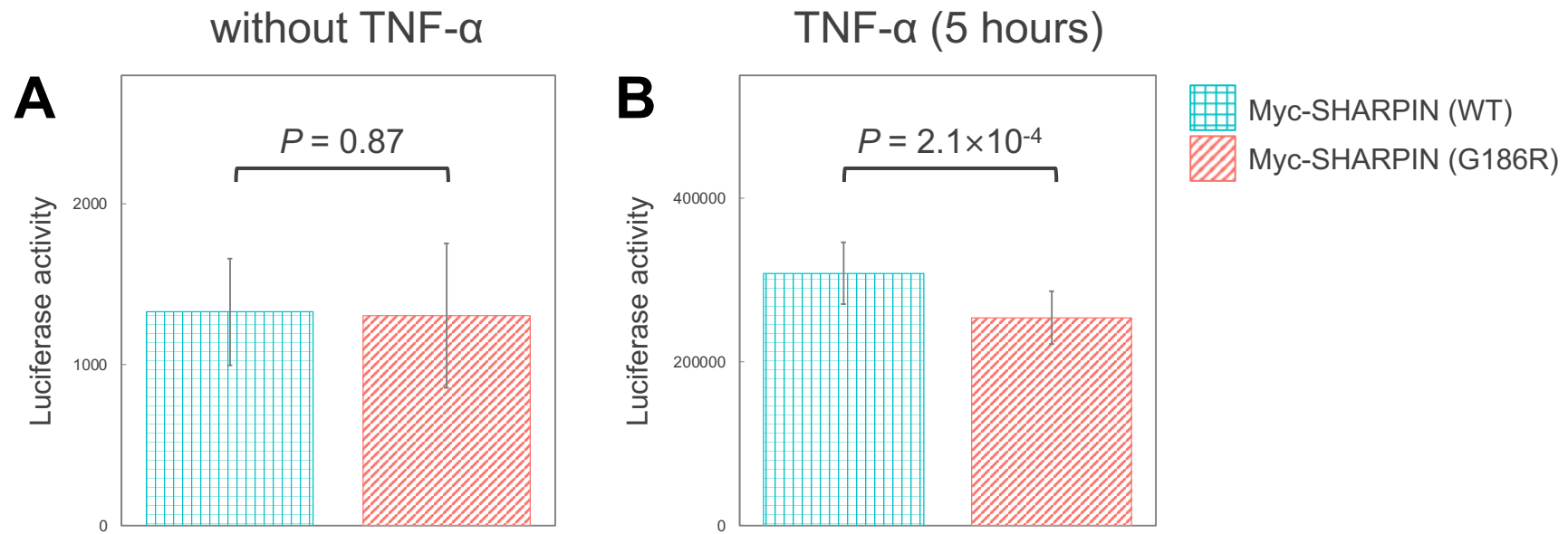

Supplement: Supplementary file 4 — Figure S3 [file 10038_2024_1224_MOESM4_ESM.pdf]
