# Supplementary material for: CRISPR/Cas9-mediated knock-in cells of the late-onset Alzheimer’s disease-risk variant, SHARPIN G186R, reveal reduced NF-κB pathway and accelerated Aβ secretion
Source: J Hum Genet. 2024 Feb 13;69(5):171–6. doi: 10.1038/s10038-024-01224-x (PMC11043039; doi:10.1038/s10038-024-01224-x)

Figure S4. Analyses for another homozygous G186R knock-in clone.

A) Sanger sequencing showing the genotypes of another G186R knock-in clone. Red arrowhead indicates the position of the target SNV and asterisk indicates the position of silent mutations. The most distant silent mutation (+19 bp from the target SNV) introduced heterozygously. **B**) Localization of G186R-type SHARPIN in the knock-in clone cell. Scale bars, 10 µm. **C**) NF-κB activity with and without TNF-α-induced activation was determined via luciferase assay (n = 5).

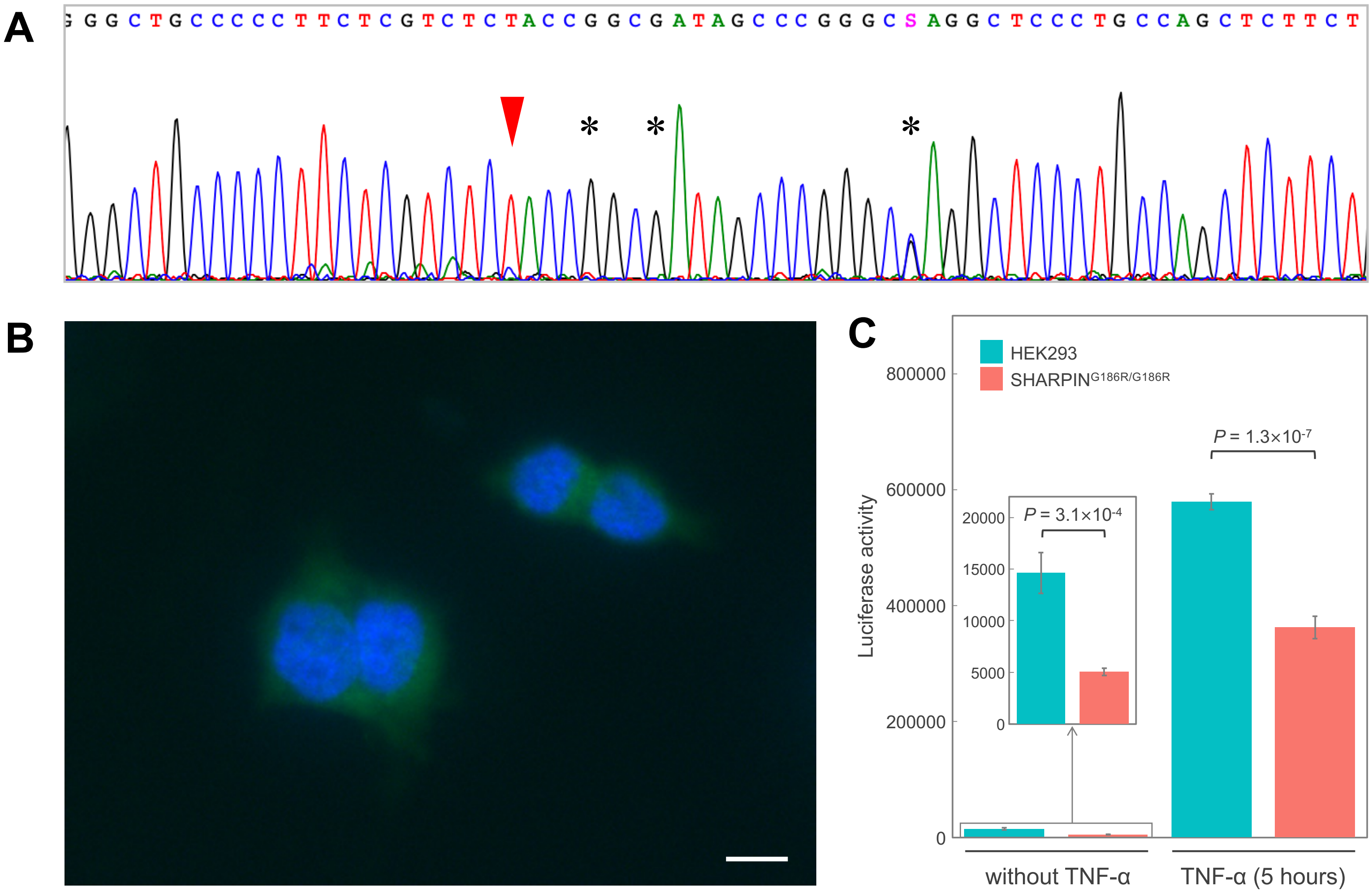

Supplement: Supplementary file 5 — Figure S4 [file 10038_2024_1224_MOESM5_ESM.pdf]
